# Supplementary material for: Increased urinary creatinine during hibernation and day roosting in the Eastern bent-winged bat (Miniopterus fuliginosus) in Korea
Source: Commun Biol. 2024 Jan 5;7:42. doi: 10.1038/s42003-023-05713-1 (PMC10770030; doi:10.1038/s42003-023-05713-1)
Supplement: Supplementary file 2 — Supplemental Information [file 42003_2023_5713_MOESM2_ESM.pdf]

Supplementary Information for:

Increased urinary creatinine during hibernation and day roosting in the Eastern bent-winged bat (*Miniopterus fuliginosus*) in Korea

Heungjin Ryu[1,2], Kodzue Kinoshita[3], Sungbae Joo[2], Yu-Seong Choi[4], Sun-Sook Kim[2]*

*Correspondence
Sun-Sook Kim
sskim108@gmail.com

This file includes:

Supplementary Figure 1 to 3
Supplementary Table 1

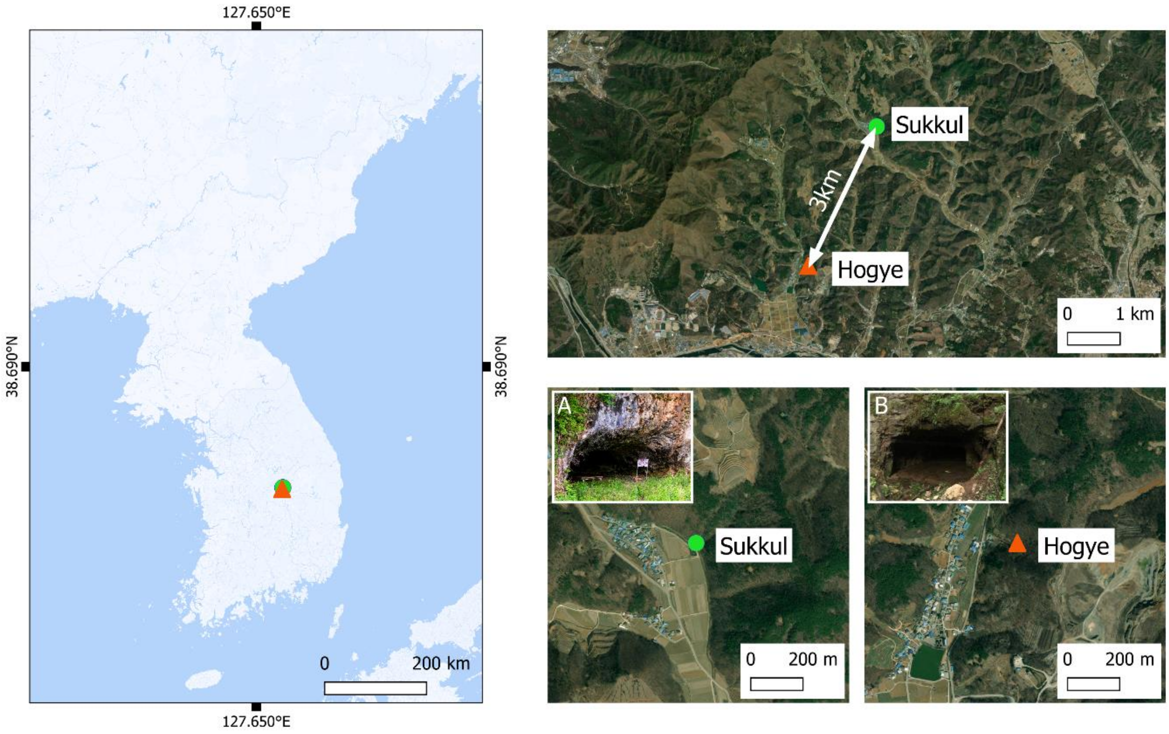

Supplementary Figure 1. The location of study sites.

Sukkul (A) is a limestone cave and Hogye (B) is an abandoned mineshaft. Bats switch between roosting sites in Hogye and Sukkul during the active period.

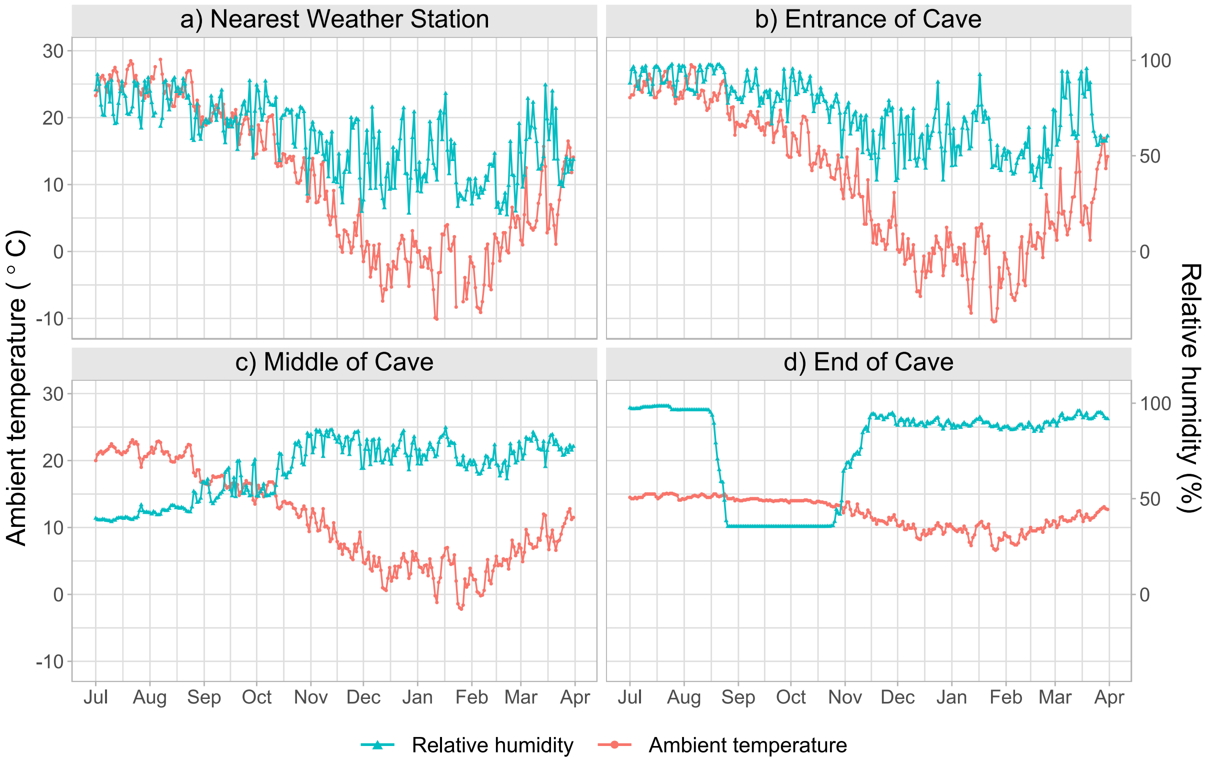

Supplementary Figure 2. Daily average relative humidity (RH) and ambient temperature (TA) at the nearest weather station (a) and different locations in Sukkul cave (b to d).

We installed loggers at the entrance (b), in the middle (c) and at the end of Sukkul (d). There might have been malfunctions in the logger installed at the end of the cave from the middle of August to November (d).

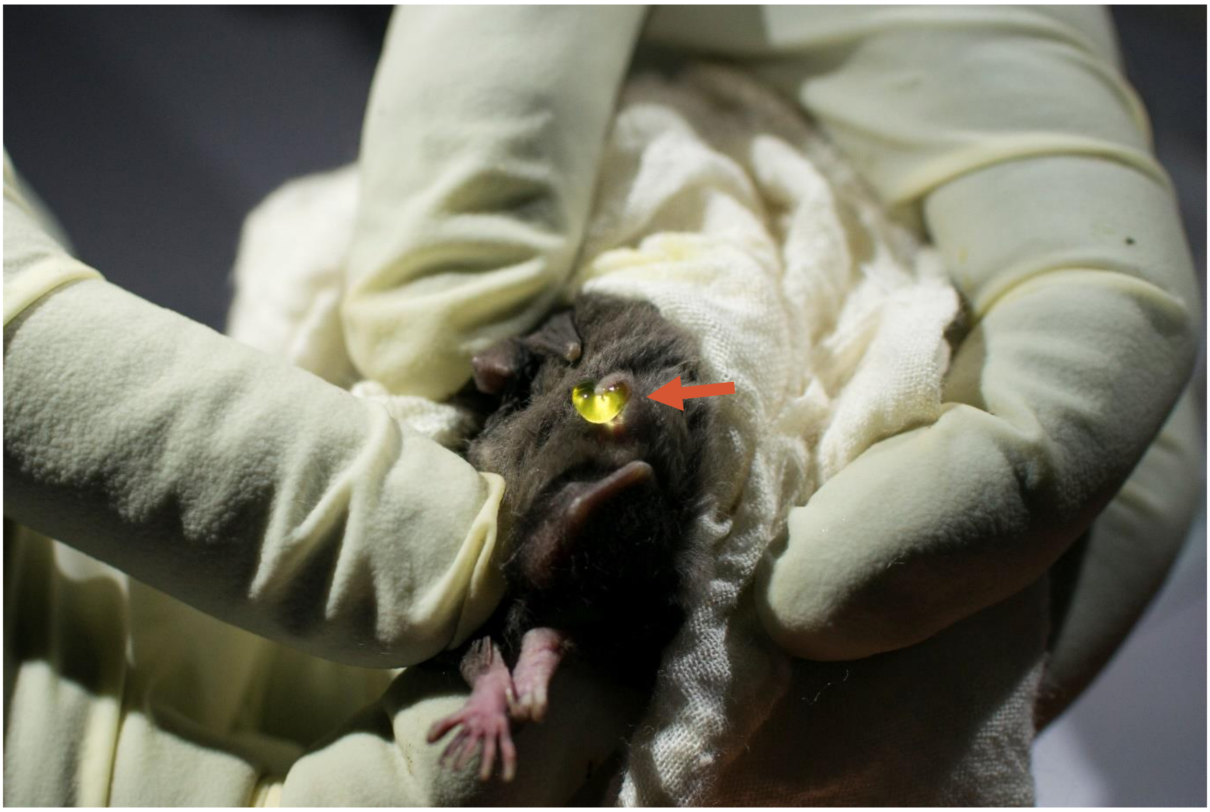

Supplementary Figure 3. Urine sample collection method.

We collected urine directly from bats right after we took out them from the cotton bag. Red arrow indicates urine from the bat.

Supplementary Table 1. Body mass and forearm length during the active season, from July to October.

The monthly average body masses of bats captured at dawn (post-feeding) were heavier than those captured at dusk (before feeding), with the exception of October. However, no similar trend was observed in the forearm length data. Note that fourteen individuals out of the 133 captured bats were not included in the table due to missing measurements for body mass and forearm length during the active season.

Months	Captured time	Number of bats	Body weight (g)	Forearm length(mm)
2017.07	Dusk (unfed)	13	15.67 ± 1.02	47.13 ± 0.70
2017.07	Dawn (fed)	13	17.52 ± 1.34	47.39 ± 1.00
2017.08	Dusk (unfed)	20	14.76 ± 0.74	47.29 ± 1.12
2017.08	Dawn (fed)	13	15.74 ± 1.00	47.14 ± 1.22
2017.09	Dusk (unfed)	23	13.74 ± 1.16	47.53 ± 0.89
2017.09	Dawn (fed)	13	15.01 ± 0.62	47.35 ± 1.01
2017.10	Dusk (unfed)	12	15.99 ± 1.18	47.25 ± 0.80
2017.10	Dawn (fed)	12	15.86 ± 1.31	47.25 ± 1.05
	Sum	119	15.33 ± 1.50	47.31 ± 0.97
