# Supplementary material for: Increased urinary creatinine during hibernation and day roosting in the Eastern bent-winged bat (Miniopterus fuliginosus) in Korea
Source: Commun Biol. 2024 Jan 5;7:42. doi: 10.1038/s42003-023-05713-1 (PMC10770030; doi:10.1038/s42003-023-05713-1)
Supplement: Supplementary file 3 — Description of Additional Supplementary Files [file 42003_2023_5713_MOESM3_ESM.docx]

Description of Additional Supplementary Files

**File name:** Supplementary Data

**Description:** Raw (source) data for the graphs and statistics in the paper
