# Supplementary material for: Increased urinary creatinine during hibernation and day roosting in the Eastern bent-winged bat (Miniopterus fuliginosus) in Korea
Source: Commun Biol. 2024 Jan 5;7:42. doi: 10.1038/s42003-023-05713-1 (PMC10770030; doi:10.1038/s42003-023-05713-1)
Supplement: Supplementary file 5 — Reporting Summary [file 42003_2023_5713_MOESM5_ESM.pdf]

nature portfolio

Corresponding author(s): Sun-Sook Kim

Last updated by author(s): Dec 6, 2023

Reporting Summary

Nature Portfolio wishes to improve the reproducibility of the work that we publish. This form provides structure for consistency and transparency in reporting. For further information on Nature Portfolio policies, see our Editorial Policies and the Editorial Policy Checklist.

Statistics

For all statistical analyses, confirm that the following items are present in the figure legend, table legend, main text, or Methods section.

n/a	Confirmed	
☐	☒	The exact sample size (*n*) for each experimental group/condition, given as a discrete number and unit of measurement
☐	☒	A statement on whether measurements were taken from distinct samples or whether the same sample was measured repeatedly
☐	☒	The statistical test(s) used AND whether they are one- or two-sided<br>*Only common tests should be described solely by name; describe more complex techniques in the Methods section.*
☐	☒	A description of all covariates tested
☐	☒	A description of any assumptions or corrections, such as tests of normality and adjustment for multiple comparisons
☐	☒	A full description of the statistical parameters including central tendency (e.g. means) or other basic estimates (e.g. regression coefficient) AND variation (e.g. standard deviation) or associated estimates of uncertainty (e.g. confidence intervals)
☐	☒	For null hypothesis testing, the test statistic (e.g. *F*, *t*, *r*) with confidence intervals, effect sizes, degrees of freedom and *P* value noted<br>*Give P values as exact values whenever suitable.*
☒	☐	For Bayesian analysis, information on the choice of priors and Markov chain Monte Carlo settings
☒	☐	For hierarchical and complex designs, identification of the appropriate level for tests and full reporting of outcomes
☒	☐	Estimates of effect sizes (e.g. Cohen's *d*, Pearson's *r*), indicating how they were calculated

Our web collection on statistics for biologists contains articles on many of the points above.

Software and code

Policy information about availability of computer code

Data collection	No software was used.
Data analysis	R 4.3.0 (R core team 2020), R packages: lme4, lmerTest, car, ggplot2, and interactions

For manuscripts utilizing custom algorithms or software that are central to the research but not yet described in published literature, software must be made available to editors and reviewers. We strongly encourage code deposition in a community repository (e.g. GitHub). See the Nature Portfolio guidelines for submitting code & software for further information.

Data

Policy information about availability of data

All manuscripts must include a data availability statement. This statement should provide the following information, where applicable:

- Accession codes, unique identifiers, or web links for publicly available datasets
- A description of any restrictions on data availability
- For clinical datasets or third party data, please ensure that the statement adheres to our policy

The datasets generated and/or analyzed during the current study are available at the Figshare Online repository (https://figshare.com/s/02bb866175c7b804c41b).

Research involving human participants, their data, or biological material

Policy information about studies with human participants or human data. See also policy information about sex, gender (identity/presentation), and sexual orientation and race, ethnicity and racism.

Reporting on sex and gender	NA
Reporting on race, ethnicity, or other socially relevant groupings	NA
Population characteristics	NA
Recruitment	NA
Ethics oversight	NA

Note that full information on the approval of the study protocol must also be provided in the manuscript.

Field-specific reporting

Please select the one below that is the best fit for your research. If you are not sure, read the appropriate sections before making your selection.

☐ Life sciences ☐ Behavioural & social sciences ☒ Ecological, evolutionary & environmental sciences

For a reference copy of the document with all sections, see nature.com/documents/nr-reporting-summary-flat.pdf

Ecological, evolutionary & environmental sciences study design

All studies must disclose on these points even when the disclosure is negative.

Study description	Urine and body size analysis on the Eastern bent-winged bats in South Korea during the active and hibernation seasons.
Research sample	A wild population of Eastern bent-winged bats living in a natural cave and mineshaft at Mungyeong, South Korea.
Sampling strategy	We conducted urine sampling and measured body sizes after hand-capturing the bats using mist nets. We tried to collect urine samples from all captured bats but if they did not urinate, we released them. Further details can be found in the Results and Methods sections.
Data collection	All authors collected urine samples in the field except KK. However, KK analyzed urine samples with HR.
Timing and spatial scale	We collected data from July 2017 to March 2018. Further details will be found in Results and Methods.
Data exclusions	We excluded data that showed less than 0 or minus concentrations or intra-assay CV over 50% from the acetoacetate assays for the figure (Fig. 5). However, the excluded data points for the figure are presented in the raw data we provided (https://figshare.com/s/02bb866175c7b804c41b).
Reproducibility	All urine analyses were conducted using double tests, and all intra-assay CVs were calculated.
Randomization	Not applicable as we could not randomly capture wild bats.
Blinding	We used all urine samples we collected for the study so blinding was not a concern.

Did the study involve field work? ☒ Yes ☐ No

Field work, collection and transport

Field conditions	A natural limestone cave and an abandoned mineshaft in the temperate zone in Korea (Supplementary Figure 1). Daily average temperatures and relative humidities are provided in Supplementary Figure 2.
Location	Mungyeong, South Korea (Supplementary Figure 1)
Access & import/export	We used our cars to get there. Urine samples were kept in a cooler with ice while we transferred them to our lab.

Disturbance	As we were handling wild bats, they might have been disturbed. Therefore, we conducted sampling twice a month during the active season and only once a month during the hibernation season.

Reporting for specific materials, systems and methods

We require information from authors about some types of materials, experimental systems and methods used in many studies. Here, indicate whether each material, system or method listed is relevant to your study. If you are not sure if a list item applies to your research, read the appropriate section before selecting a response.

Materials & experimental systems

n/a	Involved in the study
☒	☐ Antibodies
☒	☐ Eukaryotic cell lines
☒	☐ Palaeontology and archaeology
☐	☒ Animals and other organisms
☒	☐ Clinical data
☒	☐ Dual use research of concern
☒	☐ Plants

Methods

n/a	Involved in the study
☒	☐ ChIP-seq
☒	☐ Flow cytometry
☒	☐ MRI-based neuroimaging

Animals and other research organisms

Policy information about studies involving animals; ARRIVE guidelines recommended for reporting animal research, and Sex and Gender in Research

Laboratory animals	NA
Wild animals	We captured wild bats for 1 hour. We conducted urine sampling and body measurements on the captured animals. We released them at the field site right after the measurement and urine sample collection.
Reporting on sex	No sex differences were found in the study.
Field-collected samples	Urine samples that were transferred to the lab were kept in a deep freezer at -80 degrees Celsius.
Ethics oversight	Research Planning Review Committee of the National Institute of Ecology.

Note that full information on the approval of the study protocol must also be provided in the manuscript.
